# Design and Investigation of a High-Performance Quartz-Based SAW Temperature Sensor

**DOI:** 10.3390/mi15111349

**Published:** 2024-10-31

**Authors:** Jianfei Jiang

**Affiliations:** College of Biomedical Engineering & Instrument Science, Zhejiang University, Hangzhou 310027, China; jiangjianfei@zju.edu.cn

**Keywords:** surface acoustic wave, temperature sensor, coupling-of-modes, quartz

## Abstract

In this work, a surface acoustic wave (SAW) temperature sensor based on a quartz substrate was designed and investigated. Employing the Coupling-of-Modes (COM) model, a detailed analysis was conducted on the effects of the number of interdigital transducers (IDTs), the number of reflectors, and their spacing on the performance of the SAW device. The impact of the transversal mode of quartz SAWs on the device was subsequently examined using the finite element method (FEM). The simulation results indicate that optimizing these structural parameters significantly enhances the sensor’s sensitivity and frequency stability. SAW devices with optimal structural parameters were fabricated, and their resonant frequencies were tested across a temperature range of 25–150 °C. Experimental results demonstrate that the SAW temperature sensor maintains high performance stability and data reliability throughout the entire temperature range, achieving a Bode-Q of 7700. Furthermore, the sensor exhibits excellent linearity and repeatability. An analysis of the sensor’s response under varying temperature conditions reveals a significant temperature dependency on its Temperature Coefficient of Frequency (TCF). This feature suggests that the sensor possesses potential advantages for applications in industrial process control and environmental monitoring.

## 1. Introduction

SAW sensors, based on piezoelectric effect technology, have found wide applications in fields such as the Internet of Things (IoT) [1] and industrial automation [2] due to their high sensitivity, rapid response [3,4], and ease of miniaturization. SAW sensors exploit the propagation characteristics of surface acoustic waves on piezoelectric materials to detect changes in environmental parameters by observing variations in frequency or loss. Among them, quartz substrate-based SAW temperature sensors have become a research focus due to their excellent temperature stability and low-loss properties [5].

Despite the notable advantages of SAW sensors for temperature detection, several challenges persist in practical applications. Firstly, the stability and accuracy of the sensors are constrained by the stability of materials and manufacturing processes [6,7]. Traditional SAW sensor structures frequently encounter transversal mode interferences [8], which adversely affect sensor performance and increase measurement errors. To mitigate this issue, researchers have proposed several methods to suppress transversal modes, such as introducing dummy fingers or double busbar designs [9]; however, these methods necessitate further validation and optimization in practical applications. Additionally, optimizing structural parameters plays a crucial role in enhancing the performance of SAW sensors. For instance, Yudi Xie’s study demonstrated that adjusting the number of IDTs and reflectors can significantly improve sensor sensitivity [10]. Several studies have explored the operating temperature ranges of SAW sensors. For example, Xu Gao et al. tested their sensor across a range of −30~100 °C [11], while Yong Pan et al. reported stable performance within the 15~90 °C range [12]. These findings provide important context for the present work, where the sensor’s performance was evaluated within a temperature range of 25~150 °C.

Current simulations of SAW devices predominantly rely on the COM model and FEM, each with its own advantages and disadvantages. Commonly used COM models are largely based on Plessky’s two-parameter model and Hashimoto’s STW-SAW model, with various adjustments [13,14,15,16]. While the COM model offers faster simulation speeds, its accuracy is constrained by the settings of the COM parameters, which require extensive experimental data to derive precise values. FEM simulations are more accurate but are limited by mesh constraints, allowing only simplified SAW device models to be simulated, and are relatively slow. In 2016, Plessky introduced the hierarchical cascading technique (HCT), which reduces the computational load of large models by eliminating internal degrees of freedom through boundary cascading [17]. Subsequently, Xiyi Li suggested that HCT computation speeds could be enhanced using GPUs [18]. However, even with this approach, the computation speed of the COM model remains several orders of magnitude faster than that of the FEM.

This paper presents a comprehensive study and design of a quartz substrate-based SAW temperature sensor using both the COM model and the FEM. Initially, the COM model was utilized to simulate and analyze the effects of various structural parameters, including the number of IDTs, the number of reflectors, and their spacing, on the performance of the SAW device. Additionally, the temperature characteristics of the SAW device were simulated. Subsequently, FEM simulations were performed on a 3D periodic model of the SAW device to analyze the transversal modes of the quartz SAW device. Based on these simulations, SAW devices with optimal structural parameters were fabricated, and their resonant frequencies were tested at different temperatures (25~150 °C) to evaluate the performance stability and data reliability of the sensor.

This research proposes a design scheme for a SAW temperature sensor with high performance stability and data reliability, providing a reference for the future design of high-precision and high-stability temperature sensors.

## 2. Theoretical Analysis

In this section, we conduct a theoretical analysis of SAW devices. Firstly, FEM and COM models of devices used in simulation are introduced. Then, the influence of key structural parameters on the performance of SAW resonator is discussed. Finally, we study the temperature characteristics of the sensor, which provides a theoretical basis for the subsequent experimental verification and optimization.

### 2.1. Theoretical Model

**Figure 1:**
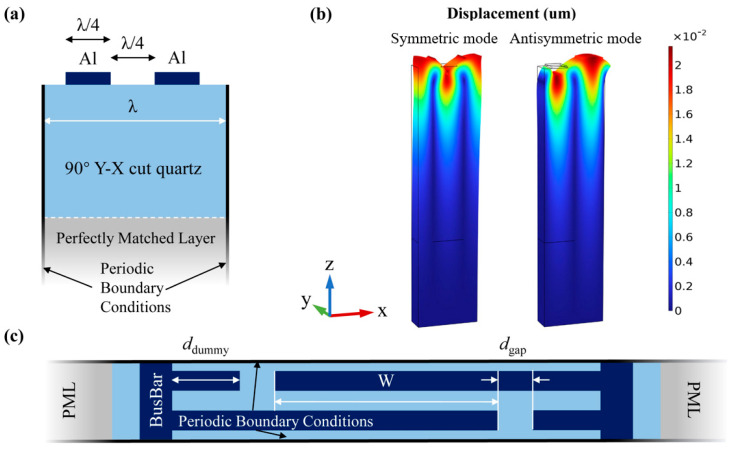
(**a**) Schematic diagram of the 2D periodic model of the SAW device; (**b**) mode shapes of the symmetric and antisymmetric modes of the Rayleigh wave; (**c**) top view of the 3D periodic model of the SAW device.

The simulation work in this study is primarily carried out using the COM model, utilizing the periodic FEM to extract accurate COM parameters [19]. Initially, a 2D periodic model of a pair of IDTs is established using COMSOL, with the specific structure shown in Figure 1a. A 90° Y-X cut quartz is used as the piezoelectric material due to its optimized non-zero temperature coefficient, making it suitable for temperature sensing, and aluminum (Al) is employed as the electrode. The wavelength of the surface acoustic wave (SAW) is fixed at 7.2 μm. In COMSOL, a rotating coordinate system with a z-x-z rotation sequence is used to define the orientation, with the 90° Y-X cut Euler angles set to (0, 90°, 0). Periodic boundary conditions are applied to left and right sides of the model to simulate an infinitely long aperture and an infinite amount of IDTs. A perfectly matched layer is introduced at the bottom of the model to simulate sufficiently thick substrate.

For the model in Figure 1a, two different configurations were applied to the electrodes: one with one electrode connected to a terminal and the other grounded, and another with both electrodes set to floating potential. By studying the Eigenfrequencies, the open-circuit and short-circuit bandgap frequencies, as well as the corresponding static capacitance values of the model, were extracted. Using these values and Equation (1), three parameters for the COM model can be obtained [20]. Figure 1b shows the mode shapes of the SAW device at the lower ($f_{sc-}$) and upper ($f_{sc+}$) edges of the stopband.

(1) $$\left\{\begin{array}{c} \;v=\frac{\lambda_{0}\left(f_{sc+}+f_{sc-}\right)}{2}\\ \left|\kappa \right|\lambda_{0}=2\pi \frac{f_{sc+}-f_{sc-}}{f_{sc+}+f_{sc-}}\\ \left|\alpha_{n}\right|=\sqrt{\omega C_{n}\lambda \pi (\frac{f_{oc+}+f_{oc-}}{f_{sc+}+f_{sc-}}-1)} \end{array}\right.$$

Meanwhile, SAW devices using conventional IDT structures exhibit some transversal parasitic modes. In this study, a 3D periodic model of the quartz SAW was established using COMSOL to simulate the impact of these transversal modes on performance. A simplified top view of the model is shown in Figure 1c.

### 2.2. Parameter Design of SAW Sensor

**Figure 2:**
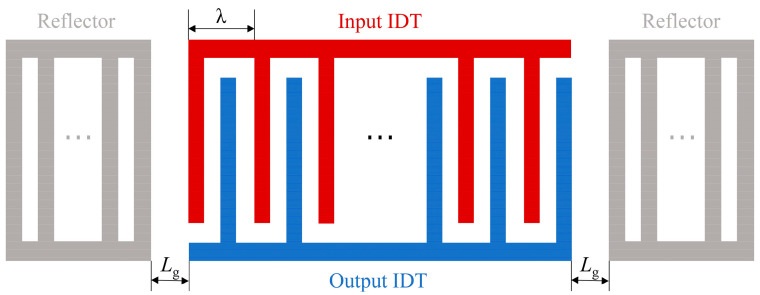
Top view of the complete SAW resonator structure.

The metal electrode layer of the SAW resonator consists of the IDT and reflectors. A top view of the complete device is shown in Figure 2. The number of IDTs (NI), the number of reflectors (NR), and the distance between them are critical factors influencing the device’s performance. In this paper, Lg is used to denote the distance from the edge of the IDT to the edge of the reflector. Since the total number of IDTs and reflectors in a SAW device usually exceeds one hundred pairs, finite element simulations of such large models require substantial computational resources and time. However, for the COM model, as long as the input parameters are sufficiently accurate, it can achieve high accuracy while maintaining efficient computational speed. Therefore, this study uses the COM model to simulate the effects of NI, NR, and Lg on the SAW device.

To reduce the energy loss caused by transversal modes in the device, this study employs the most common structure for eliminating transversal modes—the dummy finger structure [21]. By adding a segment of dummy fingers at the busbar, an energy barrier is formed, concentrating more energy in the aperture region. The transversal modes of the quartz SAW were simulated using the finite element method, and the impact of the dummy length on the transversal modes was analyzed. Some other parameters for the SAW were set as follows: the duty cycle of the interdigital electrodes was set to the common value of 0.5, and the electrode thickness was set to 150 nm.

The quality factor (Q) is a critical parameter for assessing the performance of a resonator, as it quantifies the balance between energy stored and energy dissipated during each oscillation cycle. A higher Q value indicates lower energy loss per cycle, leading to reduced dissipation and more stable resonance. This property directly translates to enhanced device performance, particularly in applications requiring high precision, such as sensing and signal processing, where frequency stability and sensitivity are paramount. For wireless sensor applications, achieving optimal performance requires the SAW device to possess a high Q factor and proper impedance matching with the wireless reader. The device’s performance is heavily influenced by the geometric parameters of the interdigital transducers (IDTs), such as the number of IDTs/reflectors and the aperture length. Establishing appropriate optimization criteria is essential for a quantitative evaluation of the SAW device’s performance. This paper systematically analyzes the variation of Bode-Q across different structural configurations, providing a comprehensive understanding of how these parameters affect overall device functionality. The formula used to calculate Bode-Q is as follows [22]:

(2) $$Q_{Bode}=\frac{\omega |S_{11}|group\_delay(S11)}{1-{\left|S_{11}\right|}^{2}}$$

where ω is the angular frequency and $\left|S_{11}\right|$ and group_delay(S11) are the amplitude of the reflection coefficient and the group delay, respectively.

### 2.3. Theory of Sensor Temperature Characteristics

Simulations were conducted to study the impact of temperature effects on the device’s resonant frequency. The principle of the SAW temperature sensor is based on the fact that changes in the ambient temperature lead to changes in the parameters of the piezoelectric material. This change results in variations in the acoustic velocity within the material, consequently causing changes in the resonant frequency of the SAW device. By monitoring these frequency changes, the external environment’s temperature can be detected. The variation in acoustic velocity in piezoelectric crystals with temperature is mainly due to changes in the material’s elastic matrix and density. For the elastic matrix, if an element $c_{ij}$ has a value of $c_{ij}\left(T_{0}\right)$ at the initial temperature T0 (usually 25 °C), the value of this element at temperature T is given by Equation (3):

(3) $$c_{ij}\left(T\right)=c_{ij}\left(T_{0}\right)\left[1+\alpha_{ij}^{c_{1}}\left(T-T_{0}\right)+\alpha_{ij}^{c_{2}}{\left(T-T_{0}\right)}^{2}+\cdots \right];i,j=1,2,\ldots ,6$$

where $\alpha_{ij}^{c_{1}}$ and $\alpha_{ij}^{c_{2}}$ are the first- and second-order thermal coefficients of the respective elasticity matrix. Similarly, at temperature *T*, the expression for the material’s density $\rho \left(T\right)$ is as follows:

(4) $$\rho \left(T\right)=\rho \left(T_{0}\right)\left[1+\alpha_{\rho }^{1}\left(T-T_{0}\right)+\alpha_{\rho }^{2}{\left(T-T_{0}\right)}^{2}+\cdots \right]$$

where $\alpha_{\rho }^{1}$ and $\alpha_{\rho }^{2}$ are the first- and second-order thermal coefficients of density, respectively. The trend in material’s elastic matrix and density with temperature variations were represented using the aforementioned equations in this work. The relevant temperature coefficients for quartz are listed in Table 1.

For the metallic material Al, temperature primarily affects its Young’s modulus. The expression for the variation of Al’s Young’s modulus with temperature is given by Equation (5) [23]:

(5) $$E\left(T\right)=-4\times 10^{7}\times T+8\times 10^{10}$$

The calculation formula for the temperature coefficient (TCF) is shown below. It is an important metric for evaluating SAW temperature sensors, where $f_{0}$ is the initial frequency, ∆T is the temperature change, and ∆f is the change in resonant frequency corresponding to the temperature change ∆T.

(6) $$TCF=\frac{1}{f_{0}}\frac{∆f}{∆T}$$

**Table 1:** Temperature-dependent properties and coefficients for quartz [24].

Variables	Properties (at 25 °C) $c_{ij}\;(GPa)$	Temperature Coefficient	
		$\alpha_{ij}^{c_{1}}\;(10^{-4}/{}^{\circ}C)$	$\alpha_{ij}^{c_{2}}\;(10^{-7}/{}^{\circ}C)$
$c_{11}$	86.7	−0.443	-
$c_{12}$	7.0	−26.9	-
$c_{13}$	11.9	−5.5	-
$c_{14}$	−17.9	1.17	-
$c_{33}$	107.2	−1.6	-
$c_{44}$	57.9	−1.754	-
		$\alpha_{ij}^{T_{1}}\;(10^{-6}/{}^{\circ}C)$	$\alpha_{ij}^{T_{2}}\;(10^{-9}/{{}^{\circ}C}^{2})$
$\alpha_{xx},\;\alpha_{yy}$		13.65	10.02
$\alpha_{zz}$		7.5	8.0
	$\rho \;(Kg/m^{3})$	$\alpha_{\rho }^{1}\;(10^{-6}/{}^{\circ}C)$	$\alpha_{\rho }^{2}\;(10^{-9}/{{}^{\circ}C}^{2})$
ρ	2650	−34.92	−15.9

## 3. Simulation Result Analysis

The structural design of SAW resonators critically impacts their performance. To find the design parameters that match a ~435 MHz resonator, this work will thoroughly analyze the effects of various structural designs on SAW resonators to optimize their performance. The finite element simulations were performed under ideal conditions, with no losses considered. In the COM simulation model, the parameter *γ* was introduced to represent the propagation loss of acoustic waves during transmission. The propagation loss increases with frequency, and, in this study, the propagation loss is expressed as follows:

(7) $$\gamma =2.24f^{2}+0.42f\;(dB/\mu s)$$

Then, using Equation (1), other COM parameter values can be calculated. Table 2 provides the COM parameters obtained for 90° Y-X quartz with 150 nm Al electrodes based on the calculations.

### 3.1. Simulation of NI and NR

**Figure 3:**
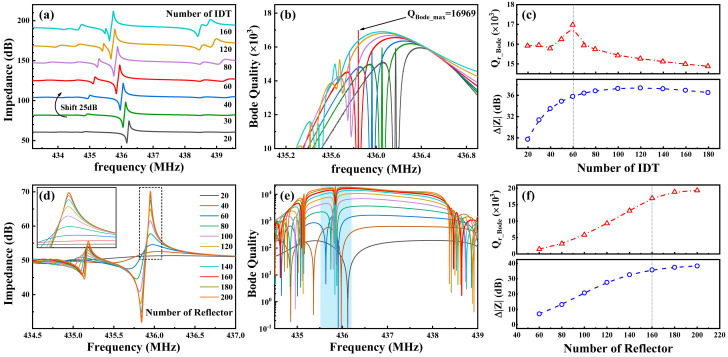
Simulation results for SAW resonators with different NI: (**a**) impedance curves, (**b**) Bode-Q curves, and (**c**) Bode-Q values (top) and impedance variation values (bottom) at the resonance point; for SAW resonators with different NR: (**d**) impedance curves, (**e**) Bode-Q curves, and (**f**) Bode-Q values (top) and impedance variation values (bottom) at the resonance point.

The number of IDTs (NI) significantly impacts the resonator performance and Q value: if NI is too small, resonance modes may not be excited, whereas if NI is too large, numerous reflections between the IDTs can occur, leading to the formation of spurious modes. First, other structural parameters were kept constant while varying NI from 20 pairs to 160 pairs, in increments of 10. The impedance curves corresponding to different numbers of IDT pairs, obtained from COM model simulations, are shown in Figure 3a. For ease of comparison, the impedance curves in Figure 3a are sequentially offset by 25 dB. The lower part of Figure 3c corresponds to the impedance variation values (maximum impedance minus minimum impedance at resonance) for different NI values. The simulation results confirm the earlier hypothesis. Figure 3b shows the Bode-Q curves of the resonator for different NI values, while the upper part of Figure 3c illustrates the Q values at the resonance frequency. By observing these two figures, it can be seen that when NI is small, the quality factor of the resonator increases with the number of pairs. However, beyond a certain threshold (NI = 60), the quality factor starts to decline. At NI = 60, the resonator’s Bode-Q reaches a maximum value of 16,969, and the parasitic modes do not significantly affect the primary mode.

In resonators, reflectors act as Bragg reflection layers that reflect surface acoustic waves back to the IDT region, reducing energy loss and achieving higher Q values. In this simulation, the NI was fixed at 60, while the number of reflector pairs (NR) varied from 20 to 200. Figure 3d,e shows the impedance curves and Bode-Q curves, respectively, for different NR values, with the *y*-axis in Figure 3e presented in a logarithmic scale. From the impedance variation values in Figure 3d,f, it is clear that when the NR is less than 40, resonance is barely observed. As the NR increases, resonance becomes more pronounced, and when the NR exceeds 160, impedance variation gradually stabilizes. Similarly, the Q value variation curves at the resonance point in Figure 3e,f show that the increase in Bode Q also tends to level off. Therefore, NR = 160 was chosen as the final device parameter, ensuring a high Q value for the resonator while saving space and reducing fabrication complexity.

### 3.2. Simulation of Distance Between IDT and Reflector

The SAW reflector grid can only achieve optimal reflection for acoustic waves that meet the Bragg condition, ensuring extremely low energy loss. This action requires that the incident wave and the reflected wave satisfy the standing wave resonance condition, which is determined by the wavelength of both the IDT and the reflector grid, as well as the distance between them. In this study, the IDT and the reflector grid have the same wavelength. Therefore, a simulation study was conducted on the distance between them to determine the most advantageous structure for the device.

**Figure 4:**
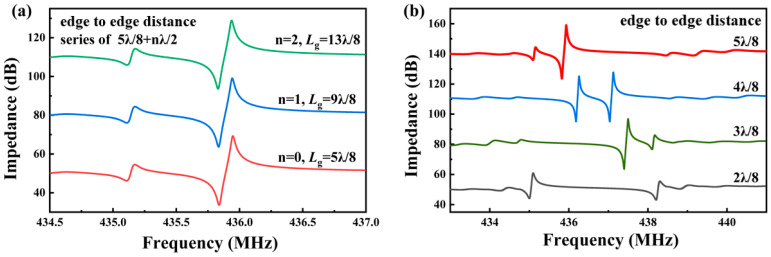
Simulated impedance curves corresponding to ***L***_g_ for (**a**) λ/8 + nλ/2 (n = 0, 1, 2) and (**b**) λ/4 + nλ/8 (n = 0, 1, 2, 3).

Using the parameters obtained from previous simulations, the distance between the IDT and the reflector grid was varied from λ/4 to 13λ/8, with a step size of λ/8. Figure 4a shows the impedance curves for distances of 5λ/8 + nλ/2 (n = 0, 1, 2), with the data offset for clarity. The distances differ by λ/2, resulting in the same phase for the round-trip travel of the acoustic wave. Consequently, the impedance curves of the devices are almost identical. However, since larger distances result in greater energy loss along the propagation path, the smallest distance should be chosen. It is noted that any Lg differing by nλ/2 follows this pattern, and the 5λ/8 series is chosen for clearer waveform comparison.

Figure 4b shows the resonance curves for an Lg of λ/4 + nλ/8 (n = 0, 1, 2, 3). Unlike traditional resonators, when Lg is λ/4, the resonator exhibits two weak resonance modes with minimal impedance variation. At an Lg of λ/2, two relatively strong resonance modes are observed, but having two modes can complicate the reader’s differentiation process when used as a wireless sensor. At an Lg of 3λ/8 and 5λ/8, a relatively weak parasitic mode is observed on the right and left sides of the main mode, respectively. Comparing the impact of the parasitic modes on the main mode, the parasitic mode at Lg = 5λ/8 has a slightly smaller impedance variation, thus Lg = 5λ/8 is chosen for the final device design.

Although the simulated optimal Lg is only applicable to quartz piezoelectric materials, it can broaden the optimization approach for future research on other devices.

### 3.3. Transversal Modal Analysis

**Figure 5:**
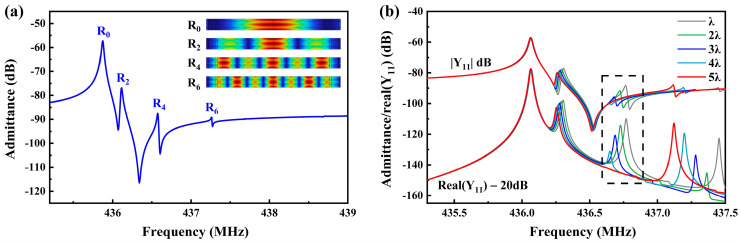
(**a**) Admittance curve of the SAW without the dummy structure; (**b**) admittance and conductance curves of the SAW device with different dummy length designs.

The impedance curve of the conventional interdigital structure was obtained using finite element method simulation on the 3D periodic model shown in Figure 1c, as illustrated in Figure 5a. It can be observed that this structure exhibits four distinct resonant modes within the simulated frequency range. The mode shapes corresponding to the resonant frequencies from low to high were marked at the top right of Figure 5a. Based on previous research, the excitation efficiency of the nth-order mode can be calculated as follows [25]:

(8) $$\eta_{n}=\left\{\begin{array}{c} \frac{\sqrt{2\;}\sin{(\beta }_{y}W_{y}/2)/(\beta_{y}W_{y}/2)\;}{\sqrt{1+\cot{(\beta }_{y}W_{y}/2)/(\beta_{y}W_{y}/2)}}\;\;\;\;\;\;\;\;\;\;\;\;\;\;\;\;\;\;\;when\;n\;is\;even\\ 0\;\;\;\;\;\;\;\;\;\;\;\;\;\;\;\;\;\;\;\;\;\;\;\;\;\;\;\;\;\;\;\;\;\;\;\;\;\;\;\;\;\;\;\;\;\;\;\;\;\;\;\;\;\;\;\;\;\;\;\;when\;n\;is\;odd \end{array}\right.\;$$

where *β_y_* is the beam width in the y-direction, and *W_y_* is the aperture length. The excitation efficiency of odd-order transversal modes is zero, meaning they will not be excited; only even-order transversal modes will appear in the passband. Therefore, the four resonant modes from low to high frequency can be identified as the 0th-order, 2nd-order, 4th-order, and 6th-order Rayleigh modes, with the 0th-order Rayleigh mode as the primary mode. To suppress the higher-order transversal modes, it is necessary to ensure that the busbar region to the aperture region has a sufficiently long low-acoustic-velocity buffer zone or to use a piston structure or double busbar structure to create a significantly lower acoustic velocity near the gap region compared to the aperture region, thereby forming an energy barrier.

This study introduces the dummy finger structure to reduce the impact of transversal modes on SAW devices. Figure 5b shows the impedance curves corresponding to different dummy finger lengths. We can observe that, as the dummy length gradually increases, R2 and R4 modes weaken progressively. When the dummy length reaches 5λ, R4 completely disappears. Simultaneously, R6 exhibits the R4 mode shape and shows enhanced resonance, but it remains far from the anti-resonance frequency. Simulations indicate that the dummy structure effectively regulates the transversal modes. Therefore, the dummy design was incorporated into the device fabrication.

### 3.4. Simulation of Sensor Temperature Characteristics

**Figure 6:**
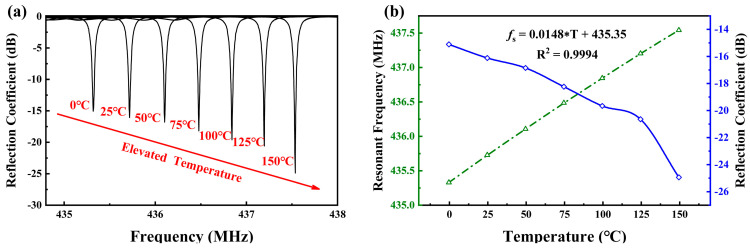
(**a**) Simulated reflection coefficient curves of the device at different temperatures; (**b**) resonant frequency and minimum reflection coefficient of the sensor at different temperatures.

Using the material parameters in Table 1 and based on COM model simulations, the characteristics of the quartz substrate-based SAW sensor were analyzed at different temperatures, ranging from 0 to 150 °C. The SAW device features 60 pairs of IDTs, 160 pairs of reflectors, and an Lg of 5λ/8. From the reflection coefficient curves of the device at different temperatures shown in Figure 6a, it can be seen that the resonant frequency increases with temperature, indicating a positive temperature coefficient. In Figure 6b, the green triangular dashed line represents the resonant frequency values at different temperatures. A linear equation fitted from the simulated data is shown in Figure 6, with an R^2^ of 0.9994, demonstrating a highly linear relationship between resonant frequency and temperature. The calculated TCF (Temperature Coefficient of Frequency) of the device is 34.0 ppm/°C, corresponding to a frequency change of 14.8 kHz/°C, which is quite significant. The blue curve in Figure 6b shows the minimum reflection coefficient at different temperatures. Within the 0–150 °C range, the minimum reflection coefficient continuously decreases as the temperature rises, resulting in lower losses at higher temperatures, making the sensor easier to detect.

## 4. Fabrication and Measurement

**Figure 7:**
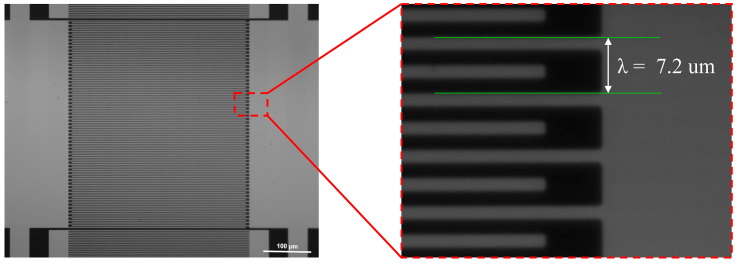
SEM image of the SAW resonator with a wavelength of 7.2 μm.

After considering the impact of various device parameters in the simulations, a SAW resonator with a wavelength of 7.2 μm was fabricated on a 90° YX-cut quartz substrate using aluminum (Al) electrode material to verify the simulation results. During the simulation of the impact of NI and NR on the resonator’s Q value, we observed that the resonator achieved its highest Q value when the number of IDTs was 60. Beyond 160 reflectors, the increase in the Q value slowed down considerably. By considering both the Q value and the device’s area utilization, we determined that using 60 IDTs and 160 reflectors would be optimal for the fabrication of the device. The designed SAW device features 60 pairs of IDTs, 160 pairs of reflectors, an Lg of 5λ/8, and an aperture length (W) of 50λ. A 150 nm Al film is deposited on the quartz through e-beam evaporation and patterned by electron beam lithography and stripping processes to form the IDT electrode and reflectors. Their frequency response characteristics were evaluated by a vector network analyzer. Figure 7 shows an SEM image of a device without a dummy finger structure, indicating that the fabricated device has minimal wavelength error.

**Figure 8:**
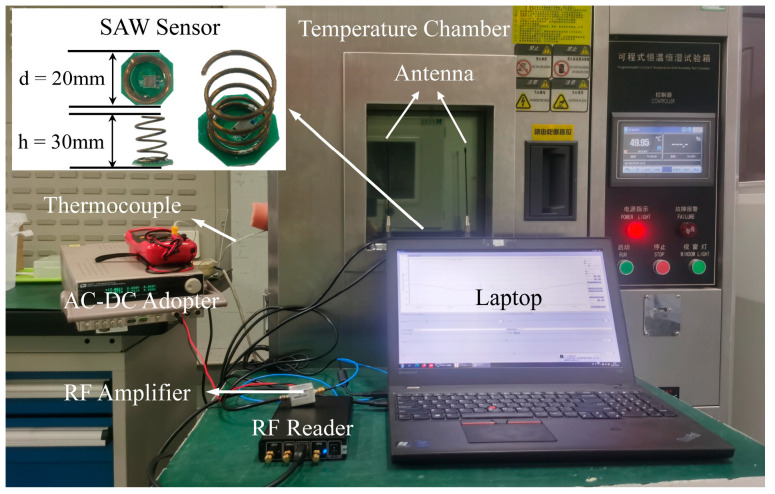
Test setup and procedure for temperature sensor evaluation.

The fabricated SAW device was connected to an antenna and placed inside a high-temperature box furnace, where the internal temperature was controlled between 25 °C and 150 °C. The device was tested using an SDR system, with a modified USRP-B210^®^ platform transmitting and receiving interrogation and response signals. Two antennas were employed due to the custom transceiver’s split TX and RX channels. A +19 dB amplifier was used to boost the TX power, ensuring adequate stimulation of the SAW device while it remained inside the temperature chamber, and the antennas were positioned outside. A laptop running Gnuradio^®^ software (GNU Radio Companion 3.7.13.5) controlled the workflow and captured the response signals. Additionally, the wireless interrogation distance of the SAW sensor was approximately 10 cm. Figure 8 illustrates the complete test setup, including the equipment and testing process. To evaluate sensor stability, the device was repeatedly heated and cooled.

**Figure 9:**
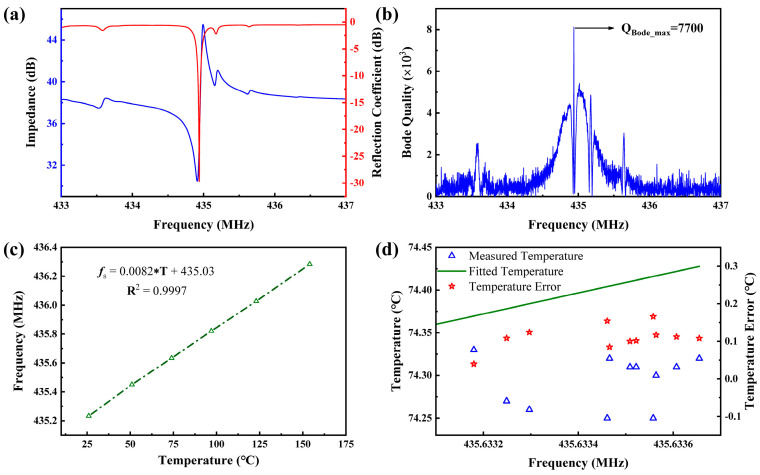
(**a**) Impedance curve and S11 test results of the SAW device at 30 °C; (**b**) Bode-Q curve of the device at 30 °C; and (**c**) variation of the sensor’s resonant frequency at different temperatures. (**d**) Error chart of temperature sensor tested multiple times.

The test results for the device at 30 °C are shown in Figure 9a, where the blue line represents the impedance curve, and the red line represents the reflection coefficient curve. The reflection coefficient at 434.94 MHz is −29.7 dB. By examining the impedance curve, it can be seen that no transversal modes appear within the resonance band due to the inclusion of the dummy finger design in the final device. While higher-order transversal spurious modes on the right side of the anti-resonance frequency are not fully suppressed, the reflection coefficient curve shows that these spurious modes do not significantly affect the primary mode.

Figure 9b presents the Bode-Q curve of the sensor at 30 °C, showing an exceptionally high Bode-Q value of 7700 at a frequency of 434.94 MHz. To test the effect of temperature on the device, the input terminal of the device was connected to an antenna and placed in a high-temperature box furnace. The resonant frequency of the device at different temperatures is illustrated in Figure 9c. As predicted by simulations, the resonant frequency of the device exhibits a linear relationship with temperature, with a linear regression R^2^ value of 0.9997, indicating a highly linear correlation between frequency and temperature. However, due to the influence of material parameters and fabrication processes, the Temperature Coefficient of Frequency (TCF) of the device is 18.9 ppm/°C, lower than the simulated value, corresponding to a frequency change of approximately 8.22 kHz/°C. Figure 9d presents the resonant frequencies measured during repeated heating and cooling cycles around 74.3 °C. The blue triangles represent the temperatures set in the temperature chamber when a specific resonant frequency was recorded. The green line corresponds to the fitted temperature-resonant frequency equation obtained in Figure 9c, while the red pentagons indicate the difference between the measured and fitted data. From Figure 9c, it is evident that the temperature deviation is within 0.2 °C, with an average error of 0.11 °C. This result demonstrates the excellent stability and reliability of this temperature sensor. Additionally, this work compares the temperature detection range and accuracy of the sensor with those reported in other studies (see Table 3). The temperature stability of the designed sensor is ±0.11 °C, and its accuracy surpasses the results reported in previous works, further confirming the superior performance of our sensor.

## 5. Conclusions

This study investigates and designs a surface acoustic wave (SAW) temperature sensor based on a quartz substrate, with a focus on analyzing the impact of its structural parameters on performance. Detailed optimization of the SAW device’s NI (number of IDTs), NR (number of reflectors), and Lg (distance between IDTs and reflectors) was conducted using the COM model. FEM simulations were employed to assess the effect of dummy structures on transversal modes. The device designed with the simulation parameters achieved an S11 minimum value of −29.7 dB and a Bode-Q of 7700, demonstrating excellent performance. Additionally, the SAW temperature sensor exhibited good linearity and stability within the temperature range of 25~150 °C, with a measured TCF of 18.9 ppm/°C. By integrating simulation and experimental validation, this study systematically optimized the design of the SAW temperature sensor. This outcome not only enhanced the sensor’s sensitivity and stability but also provided strong support for its practical applications in industrial process control and environmental monitoring. The findings offer important references for the design of high-precision, high-stability temperature sensors in the future, thereby advancing the development of SAW temperature sensing technology.

## Figures and Tables

**Table 2:** COM parameters for 90° Y-X quartz with 150 nm Al electrodes.

Parameters	Symbol	Values	Dimension (SI)
Normalized Static capacitance	$C_{n}=C\lambda_{0}/W$	49.34	pF/m
SAW Velocity	*v*	3143.8	m/s
Normalized Coupling coefficient	$\kappa_{p}=\kappa \lambda_{0}$	2.16	%
Normalized Transduction coefficient	$\alpha_{p}=\alpha_{n}\lambda_{0}$	6.56	%

**Table 3:** The accuracy comparison between the proposed sensor with similar works.

Frequency of SAW Device	Temperature Detection Range	Accuracy	References
2.45 GHz	30 °C~200 °C	±0.5 °C	[26]
429.2 MHz	25 °C~130 °C	±0.6 °C	[27]
2.4 GHz	25 °C~200 °C	±0.6 °C	[28]
434 MHz	15 °C~90 °C	±0.8 °C	[12]
433 MHz	−30 °C~100 °C	±0.2 °C	[11]
435 MHz	25 °C~150 °C	±0.11 °C	This work

## Data Availability

The original contributions presented in the study are included in the article, further inquiries can be directed to the corresponding author.
